# Supplementary material for: Diseasomics: Actionable machine interpretable disease knowledge at the point-of-care
Source: PLOS Digit Health. 2022 Oct 20;1(10):e0000128. doi: 10.1371/journal.pdig.0000128 (PMC9931276; doi:10.1371/journal.pdig.0000128)
Supplement: S1 Text — (PDF) [file pdig.0000128.s001.pdf]

Supporting Information S1 Text for Talukder AK, Schriml L, Ghosh A, Biswas R, Chakrabarti P, Haas RE. Diseasomics: Actionable Machine Interpretable Disease Knowledge at the Point-of-Care

For this work, we have used the following biomedical knowledge sources as shown in Fig 1:

1. Symptom ontology file "symp.obo" downloaded from https://github.com/DiseaseOntology/SymptomOntology on 26th January 2021.
2. Disease ontology file "doid.obo" downloaded from https://github.com/DiseaseOntology/HumanDiseaseOntology on 26th January 2021.
3. DisGenNet ontology file "curated_gene_disease_associations_tsv" downloaded from https://www.disgenet.org/downloads on 26th January 2021.
4. For HGNC mapping we have used Custom Download constructed from https://www.genenames.org/download/custom/ with HGNC Id, Approved Symbol, Approved name, and few other fields.
5. SNOMED CT downloaded from UMLS on August 26, 2019.
6. Trajectory data "41467_2014_BFncomms5022_MOESM730_ESM.xlsx" downloaded on 26th January 2021 from https://www.nature.com/articles/ncomms5022
7. Pharmacogentics data "drugLabels.tsv" from PharmGKB on 6th February 2021 from https://www.pharmgkb.org/downloads
8. Spatial Comorbidity data shared by author of [1].

References

1. Talukder AK, Sanz JB, Samajpati J. 'Precision Health': Balancing Reactive Care and Proactive Care Through the Evidence Based Knowledge Graph Constructed from Real-World Electronic Health Records, Disease Trajectories, Diseasome, and Patholome. BDA 2020. doi: 10.1007/978-3-030-66665-1_9.
