# Supplementary material for: Diseasomics: Actionable machine interpretable disease knowledge at the point-of-care
Source: PLOS Digit Health. 2022 Oct 20;1(10):e0000128. doi: 10.1371/journal.pdig.0000128 (PMC9931276; doi:10.1371/journal.pdig.0000128)
Supplement: S2 Text — (PDF) [file pdig.0000128.s002.pdf]

Supporting Information S2 Text for Talukder AK, Schriml L, Ghosh A, Biswas R, Chakrabarti P, Haas RE. Diseasomics: Actionable Machine Interpretable Disease Knowledge at the Point-of-Care

The diseasomics we constructed is both human understandable and computer interpretable. The computer interpretable diseasomics knowledge base is stored in a graph database.

This supplementary information contains screenshots of ancillary medical knowledge that supports the diseasomics to offer holistic healthcare. Because the medical knowledge in the diseasomics knowledge graph is machine interpretable, this knowledge is enhanced through controlled vocabulary with the help of *semantic* & *thematic* integration of external knowledge sources. Following screenshots show the advantage of machine interpretable actionable medical knowledge (*physicians' digital twins*) that can be used by expert and non-expert health workers alike.

```
$ MATCH p=(c:CMBD{Age:'1060069',ICD10CM:'I10'})-[r:CMBD]-(b) RETURN c.Age,c.Desc,b.ICD10CM,b.Desc
```

Table | Text | Code

c.Age	c.Desc	b.ICD10CM	b.Desc
"1060069"	"Essential (primary) hypertension"	"E66.3"	"Overweight"
"1060069"	"Essential (primary) hypertension"	"E66.01"	"Morbid (severe) obesity due to excess calories"
"1060069"	"Essential (primary) hypertension"	"D51.9"	"Vitamin B12 deficiency anemia; unspecified"
"1060069"	"Essential (primary) hypertension"	"B34.2"	"Coronavirus infection; unspecified"
"1060069"	"Essential (primary) hypertension"	"Z99.89"	"Dependence on other enabling machines and devices"
"1060069"	"Essential (primary) hypertension"	"Z98.61"	"Coronary angioplasty status"
"1060069"	"Essential (primary) [illegible]	"Z95.5"	"Presence of coronary angioplasty implant and graft"

Started streaming 660 records after 3 ms and completed after 82 ms.

Fig 1 in S2 Text: Statistically significant (p-value < 0.05) *spatial comorbidity* for male of age between 60 to 69 population (1060069). This knowledge is thematically integrated with the *diseasomics* [1].

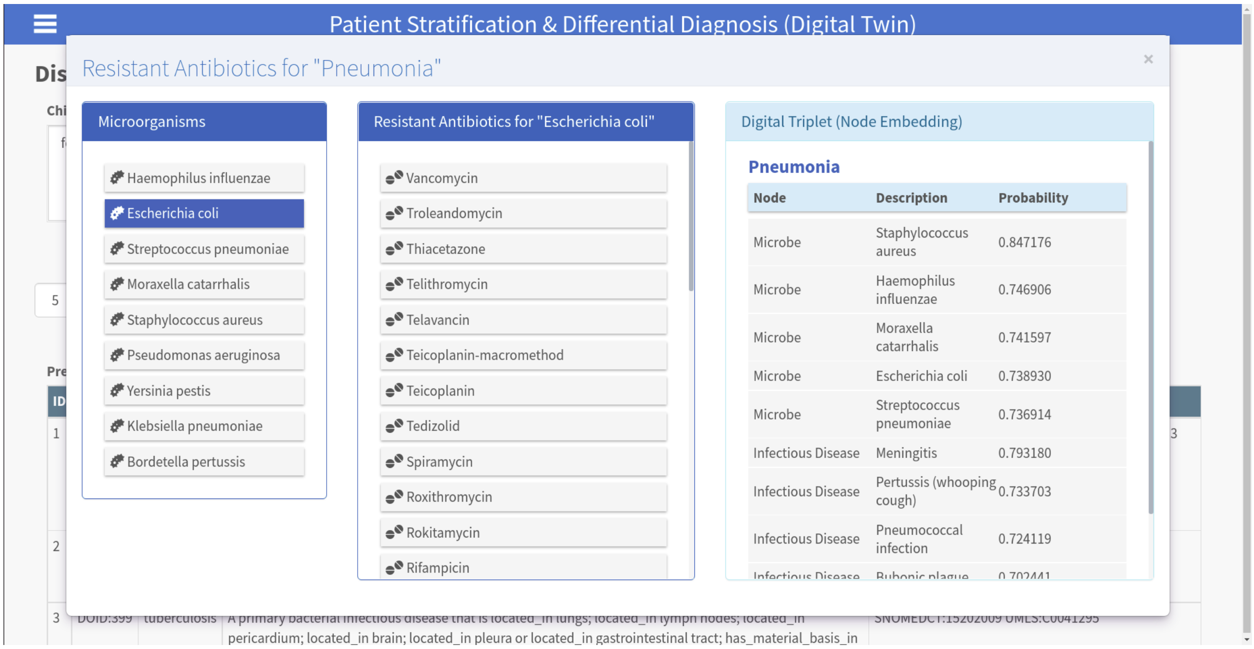

Fig 2 in S2 Text: The *resistomics* integration with the diseasomics knowledge graph. In Figure 3 first disease shown is pneumonia for co-occuring symptoms fever, night sweat, and coughing blood. When we click pneumonia (the first disease in Figure 3) in the Disease column a resistomics screen pops up as shown here. First column in the pop-up resistomics screen shows all disease causing bacteria for bacterial pneumonia. Second column in the resistomics screen shows the antibiotics that are resistant to the selected bacteria Escherichia coli in this case. The third column is the digital triplet of pneumonia. The Digital Triplet is constructed through *vector embedding* [2].

```
$ MATCH p=(n:NCIT {NCIT:'NCIT:C3081'})--(N) RETURN N.Name,N.Desc
```

N.Name	N.Desc
"Cardiac Paraganglioma"	"A benign or malignant extra-adrenal paraganglioma arising from paraganglia located in the heart. [] {http://purl.obolibrary.org/obo/NCIT_P378= NCI }"
"Heart Lymphoma"	"An extranodal lymphoma that arises from the heart and/or the pericardium. The majority of the cases are diffuse large B-cell lymphomas. Patients may present with chest pain; heart failure; pericardial effusion; arrhythmia; or syncope. [] {http://purl.obolibrary.org/obo/NCIT_P378= NCI }"
"Myocardial Neoplasm"	"A benign or malignant neoplasm that affects the myocardium. [] {http://purl.obolibrary.org/obo/NCIT_P378= NCI }"
"Epicardial Neoplasm"	"A benign or malignant neoplasm that affects the inner layer of the pericardium. [] {http://purl.obolibrary.org/obo/NCIT_P378= NCI }"
"Endocardial Neoplasm"	"A benign or malignant neoplasm that affects the endocardium. [] {http://purl.obolibrary.org/obo/NCIT_P378= NCI }"
"Pericardial Neoplasm"	"A benign or malignant neoplasm that affects the pericardium. [] {http://purl.obolibrary.org/obo/NCIT_P378= NCI }"
"Cardiac Inflammatory Myofibroblastic Tumor"	"An intermediate fibroblastic neoplasm arising from the heart. It is characterized by the presence of spindle-shaped fibroblasts and myofibroblasts; and a chronic inflammatory infiltrate composed of eosinophils; lymphocytes; and plasma cells. [] {http://purl.obolibrary.org/obo/NCIT_P378= NCI }"
"Benign Cardiac Neoplasm"	"A non-metastasizing neoplasm that arises from the heart. Representative examples include atrial myxoma; cardiac lipoma; and cardiac fibroma. [] {http://purl.obolibrary.org/obo/NCIT_P378= NCI }"
"Malignant Cardiac Neoplasm"	"A primary or metastatic malignant neoplasm involving the heart. [] {http://purl.obolibrary.org/obo/NCIT_P378= NCI }"
"Cardiovascular Neoplasm"	"A benign or malignant neoplasm that affects the heart and/or vessels. Representative examples of benign neoplasms include atrial myxoma; hemangioma; and lymphangioma. Representative examples of malignant neoplasms include pericardial malignant mesothelioma and angiosarcoma. [] {http://purl.obolibrary.org/obo/NCIT_P378= NCI }"
"Thoracic Neoplasm"	"A benign or malignant; primary or metastatic neoplasm involving the tissues of the thorax. [] {http://purl.obolibrary.org/obo/NCIT_P378= NCI }"
"Heart Disorder"	"A non-neoplastic or neoplastic disorder that affects the heart and/or the pericardium. Representative examples include endocarditis; pericarditis; atrial myxoma; cardiac myeloid sarcoma; and pericardial malignant mesothelioma. [] {http://purl.obolibrary.org/obo/NCIT_P378= NCI }"
"Cardiac Germ Cell Tumor"	"A germ cell tumor that arises within the myocardium or cardiac chambers. The reported cases have been teratomas and yolk sac tumors. []

Started streaming 14 records after 1 ms and completed after 94 ms.

Fig 3 in S2 Text: The *oncolomics* knowledge for NCIt code NCIT:C3081. In Figure 3 of the main article, the second disease in the Disease column is *heart cancer*. The CodeInfo column for heart cancer includes the NCIT code NCIT:C3081 and NCIT:C3548. Here in this figure we see all nodes with their respective names and descriptions connected to NCIT:C3081 in the oncolomics knowledge graph [3].

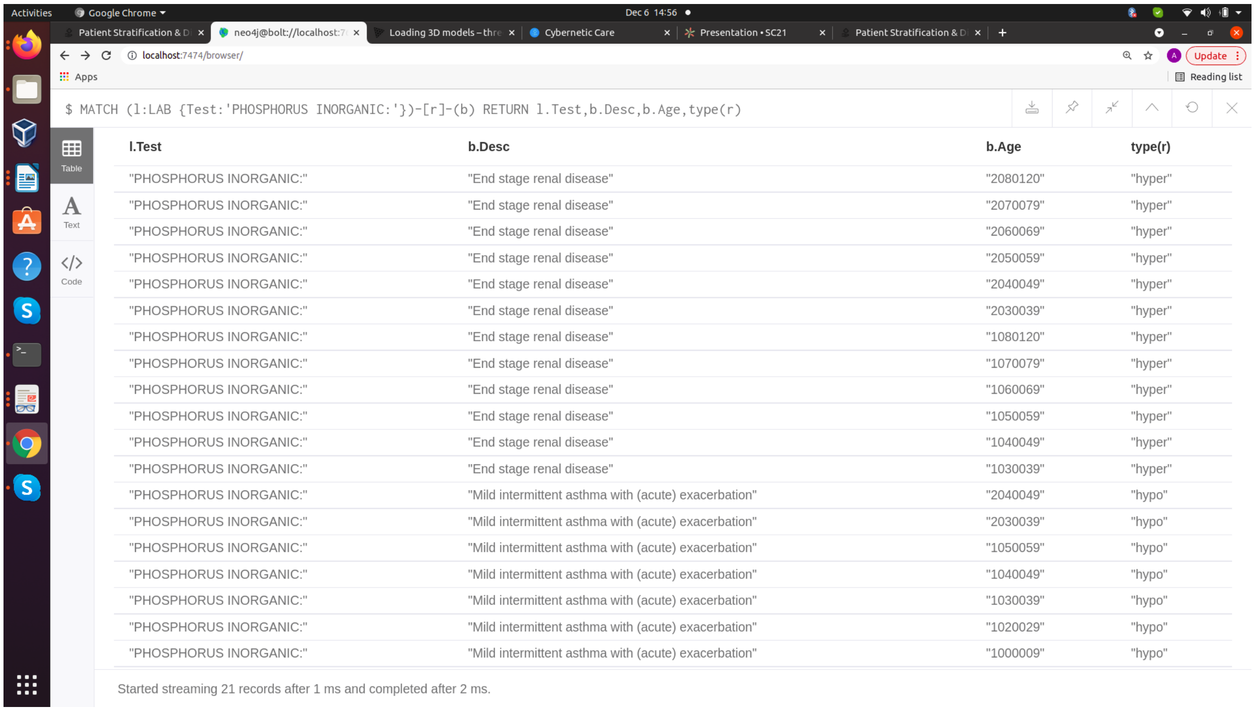

$ MATCH (l:LAB {Test:'PHOSPHORUS INORGANIC:'})-[r]-(b) RETURN l.Test,b.Desc,b.Age,type(r)

l.Test	b.Desc	b.Age	type(r)
"PHOSPHORUS INORGANIC:"	"End stage renal disease"	"2080120"	"hyper"
"PHOSPHORUS INORGANIC:"	"End stage renal disease"	"2070079"	"hyper"
"PHOSPHORUS INORGANIC:"	"End stage renal disease"	"2060069"	"hyper"
"PHOSPHORUS INORGANIC:"	"End stage renal disease"	"2050059"	"hyper"
"PHOSPHORUS INORGANIC:"	"End stage renal disease"	"2040049"	"hyper"
"PHOSPHORUS INORGANIC:"	"End stage renal disease"	"2030039"	"hyper"
"PHOSPHORUS INORGANIC:"	"End stage renal disease"	"1080120"	"hyper"
"PHOSPHORUS INORGANIC:"	"End stage renal disease"	"1070079"	"hyper"
"PHOSPHORUS INORGANIC:"	"End stage renal disease"	"1060069"	"hyper"
"PHOSPHORUS INORGANIC:"	"End stage renal disease"	"1050059"	"hyper"
"PHOSPHORUS INORGANIC:"	"End stage renal disease"	"1040049"	"hyper"
"PHOSPHORUS INORGANIC:"	"End stage renal disease"	"1030039"	"hyper"
"PHOSPHORUS INORGANIC:"	"Mild intermittent asthma with (acute) exacerbation"	"2040049"	"hypo"
"PHOSPHORUS INORGANIC:"	"Mild intermittent asthma with (acute) exacerbation"	"2030039"	"hypo"
"PHOSPHORUS INORGANIC:"	"Mild intermittent asthma with (acute) exacerbation"	"1050059"	"hypo"
"PHOSPHORUS INORGANIC:"	"Mild intermittent asthma with (acute) exacerbation"	"1040049"	"hypo"
"PHOSPHORUS INORGANIC:"	"Mild intermittent asthma with (acute) exacerbation"	"1030039"	"hypo"
"PHOSPHORUS INORGANIC:"	"Mild intermittent asthma with (acute) exacerbation"	"1020029"	"hypo"
"PHOSPHORUS INORGANIC:"	"Mild intermittent asthma with (acute) exacerbation"	"1000009"	"hypo"

Started streaming 21 records after 1 ms and completed after 2 ms.

Fig 4 in S2 Text: Semantic integration of *patholomics* with *diseasomics*. In this figure we show the pathology test with the disease description and the statistically significant age group. Here we show the hypophosphatemia and hyperphosphatemia for different age groups with associated diseases [1].

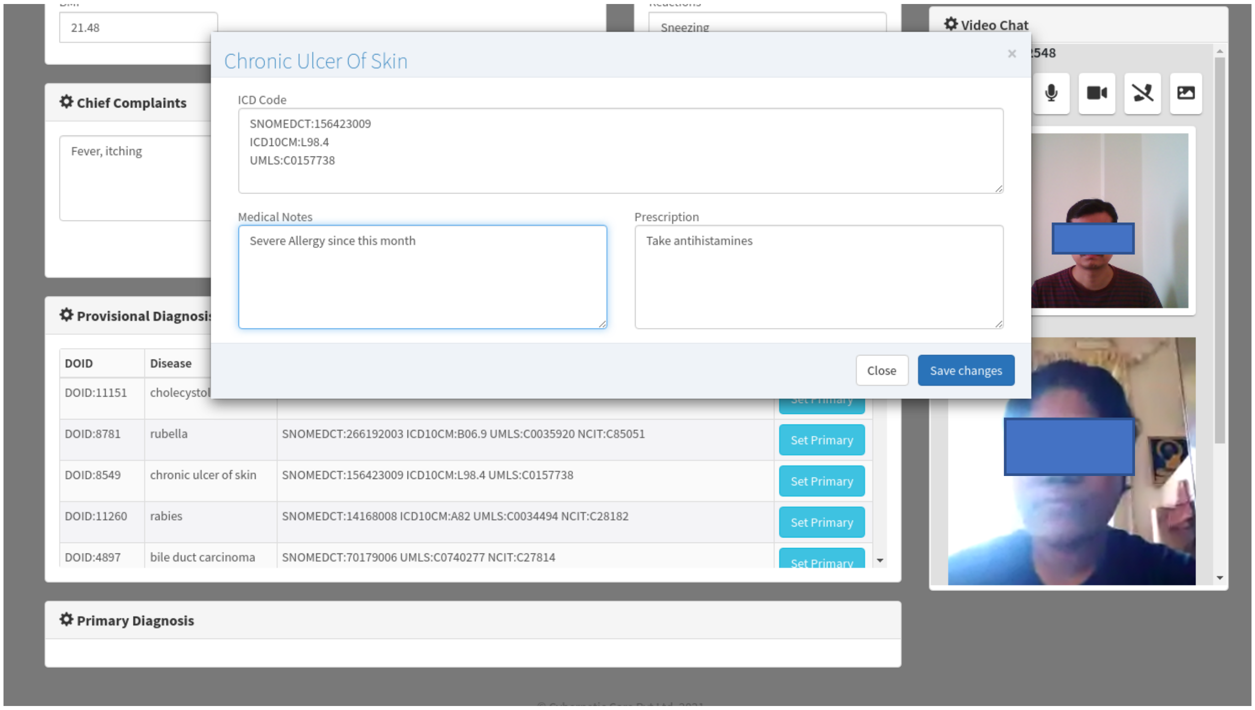

Fig 5 in S2 Text: This figure shows *diseasomics* used in *telemedicine* for remote care integrated with patient records in the EHR [4]. During a telemedicine session, the doctor and patient are having an audiovisual chat using WebRTC technology. WebRTC is ultra-low latency realtime peer-to-peer and end-to-end encrypted communication between two browsers. WebRTC is standardized by W3C and IETF and works without any plugins or downloads or any intermediate servers. In this figure we can see *differential diagnoses* from the diseasomics knowledge graph accessed through API. The care provider selects one of these diseases as the primary diagnosis. The caregiver also enters the medical notes and the prescribed medication which are saved in the EHR.

References

1. Talukder AK, Sanz JB, Samajpati J. 'Precision Health': Balancing Reactive Care and Proactive Care Through the Evidence Based Knowledge Graph Constructed from Real-World Electronic Health Records, Disease Trajectories, Diseasome, and Patholome. BDA 2020. doi: 10.1007/978-3-030-66665-1_9.
2. Talukder AK, Chakrabarti P, Chaudhuri B, Sethi T, Lodha R, Haas RE. 2AI&7D Model of Resistomics to Counter the Accelerating Antibiotic Resistance and the Medical Climate Crisis. S. N. Srirama et al. editors. BDA 2021. doi: 10.1007/978-3-030-93620-4_4.
3. Talukder AK, Haas RE. Oncolomics: Digital Twins and Digital Triplets in Cancer Care. CAFCW21: Computational Approaches for Cancer Workshop 2021. Avalable from: https://sc21.supercomputing.org/presentation/?id=ws_cafcw103&sess=sess434
4. Talukder AK, Haas RE. AIoT: AI meets IoT and Web in Smart Healthcare. In 13th ACM Web Science Conference 2020. doi: 10.1145/3462741.3466650.
