# Supplementary material for: Diseasomics: Actionable machine interpretable disease knowledge at the point-of-care
Source: PLOS Digit Health. 2022 Oct 20;1(10):e0000128. doi: 10.1371/journal.pdig.0000128 (PMC9931276; doi:10.1371/journal.pdig.0000128)
Supplement: S3 Text — (PDF) [file pdig.0000128.s003.pdf]

Supporting Information S3 Text for Talukder AK, Schriml L, Ghosh A, Biswas R, Chakrabarti P, Haas RE. Diseasomics: Actionable Machine Interpretable Disease Knowledge at the Point-of-Care

The knowledge graphs as explained in the article "Diseasomics: Actionable Machine Interpretable Disease Knowledge at the Point-of-Care" are embedded within a Unix/Linux shell script named 'NeoImportDO.sh' released in the public domain with Apache 2.0 license modified with Commons Clause. The NeoImportDO.sh is a self-sufficient shell script. The shell script will create all node files and all relationship files from the embedded data. It will create a new Neo4j graph database instance, load all nodes and edges (relationships), and finally start the Neo4j database in an automated fashion. The user needs to point the NEO4J and CSVDATA environment variables to the Neo4j home directory and a temporary working directory, respectively. Following these changes, the user runs the shell script. The user will access the diseasomics knowledge either through the Neo4j browser or the Neo4j Cypher commands API. The NeoImportDO.zip self-loading knowledge graph can be downloaded from https://zenodo.org [1] by searching for the term NeoImportDO.zip in the upper search field (https://zenodo.org/search?page=1&size=20&q=NeoImportDO.zip).

SNOMED CT being licensed data, is not added in the open domain diseasomics knowledge graph. Any healthcare provider or researcher interested in integrating SNOMED CT as described in this paper will need to deploy the Neo4j version of SNOMED CT and semantically connect the diseasomics knowledge graph with SNOMED CT. This will allow a user to navigate from symptom to disease to any SNOMED CT concept hierarchy; for example, 'Body Structure' or 'Pharmaceutical/Biological product'. The symptom-disease network is not released in the public domain but can be obtained from the authors.

References

1. NeoImportDO.zip self-loading knowledge graph. Available from https://zenodo.org/search?page=1&size=20&q=NeoImportDO.zip. doi: 10.5281/zenodo.6416938.
