# Supplementary material for: Diseasomics: Actionable machine interpretable disease knowledge at the point-of-care
Source: PLOS Digit Health. 2022 Oct 20;1(10):e0000128. doi: 10.1371/journal.pdig.0000128 (PMC9931276; doi:10.1371/journal.pdig.0000128)
Supplement: Attachment — Submitted filename: Response to Reviewers.pdf [file pdig.0000128.s006.pdf]

---------------
## Reviewer#1
---------------
**Reviewer #1:**
The paper entitled "Diseasomics: Actionable Machine Interpretable Disease Knowledge at the Point-of-Care" is an excellent presentation of the possibilities available to us through careful and thoughtful analysis of ontologies. The most important part is the exercise that presents the relationships among terms to aid cause and effect in disease discovery.
The graphic representation of disease and pharmacology and the analysis of the relationships shown is outstanding. The attempt to understand and normalize the complex terms in the UMLS under 'findings', 'signs and symptoms' is one of the best I have seen so far to put in use this complex area. Findings, signs and symptoms are complex and difficult to understand in the real world, and tend to be 'messy' as described by other authors. most informatics studies tend to discard them to avoid confusion and decrease the amount of manual annotation and work. Incorporating them in the study brings in a new level that clearly shows their importance."

**>>>> Authors' Response**
Thank you.

**Reviewer #1:**
It will be interesting to see next steps where LOINC terms are brought in the picture.

**>>>> Authors' Response**
We have plans to include LOINC in the future

---------------
## Reviewer#2
---------------
**Reviewer #2:**
The study aims to bring together various knowledge sources to help with diagnosis decisions through a knowledge graph, eventually targeting the healthcare system of low and middle income countries.

The paper is well written and explains the process of aggregating multiple knowledge sources for clinical decision support. I will leave the assessment of the technical details to the other reviewers and will point out a few concerns I have in relation to the overall study.

**>>>> Authors' Response**
Thank you

**Reviewer #2:**
- I appreciate the detailed presentation of all the information sources and the process to combine them. But, I think these could be presented in a more concise way perhaps with a big table.

**>>>> Authors' Response**
We have tried to make it as comprehensive as possible without compromising the process flow so that the readers can understand it and reproduce it for further validation

**Reviewer #2:**

- Using Wikipedia as the main source of information is very problematic. The rationale behind this choice should be explained clearly.

>>> **Authors' Response** (old reference numbering)
Earlier works used MEDLINE/PubMed as the knowledge source [26]. MEDLINE/PubMed contains advanced research knowledge that includes mainly complex diseases or diseases with complex conditions useful for tertiary care or specialized care. To overcome this challenge we used Wikipedia as our primary source of signs and symptoms that are necessary at the primary/secondary care. Wherever we found Wikipedia to be inadequate we used PubMed and literature as secondary knowledge sources curated by human experts.

**Reviewer #2:**
- The background section covers some prior work but a critical assessment of the current state of art in this information aggregation and generating a differential diagnosis is missing. For example, "The 'Symptom Ontology' provides the ontology of many symptoms but lacks the cross-references to diseases [25]. Xia et al. attempted to address this gap [26]." What should we understand from this statement, did Xia et al succeed? what did they achieve? Similarly, "Rotmensch et al. constructed a disease-symptom network of 156 diseases and 491 symptoms from emergency department patient visits [28]. Here for the first time we present 11,188 conditions that include signs, symptoms, findings, diseases, syndromes, and neoplasm with 10,579 associated diseases." Does this mean the number of conditions and diseases are the higher than any prior systems? Please describe the unique contribution of this study more clearly.

>>>> **Authors' Response**
We replaced the entire paragraph#2 in Page 3
[Original Text, old reference numbering]
One critical component in evidence-based and networked medicine [24] is the diagnosis of a disease from the patient's signs, symptoms, findings, and co-existing conditions. However, there is limited work in this area of machine interpretable knowledge. The 'Symptom Ontology' provides the ontology of many symptoms but lacks the cross-references to diseases [25]. Xia et al. attempted to address this gap [26]. Zhou et al. constructed a symptom-based human disease network of 134 diseases and 316 symptoms through the diseases and their underlying molecular interactions [27]. Rotmensch et al. constructed a disease-symptom network of 156 diseases and 491 symptoms from emergency department patient visits [28]. Here for the first time we present 11,188 conditions that include signs, symptoms, findings, diseases, syndromes, and neoplasm with 10,579 associated diseases.

We replaced the original text with new text as given below
[New Text, new reference numbering]
One critical component in evidence-based and networked medicine [28] is the diagnosis of a disease from the patient's signs, symptoms, findings, and co-existing conditions. However, there is limited work in this area of machine interpretable knowledge. The 'Symptom Ontology' provides the ontology of many symptoms but lacks the cross-references to diseases [29]. Xia et al. mined literature to identify and retrieve symptom to disease relationships and build a symptom-disease knowledge graph [30]. They identified alignment of 8,514 disease concepts from the Human Disease Ontology and symptoms from the Symptom Ontology to 15,970,134 MEDLINE/PubMed citation records [30]. Because this knowledge is mined from biomedical literature, it is biased towards complex and severe diseases, or diseases having severe conditions. Zhou et al. constructed a symptom-based human disease network of 134 diseases and 316 symptoms through the diseases and their underlying molecular interactions [31]. This network is biased towards genetic disorders. Rotmensch et al. constructed a disease-symptom network of 156

diseases and 491 symptoms from emergency department patient visits, which is biased towards emergency care [32]. The above knowledge constructed by Zhou et al. [31] and Rotmensch et al. [32] are available in the open domain, therefore we semantically integrated them with the current diseasomics knowledge graph. The 'Diseaseome tool' has improved upon previous disease-symptom relation mining work by: (1) utilizing the DO's expanded disease classification of 10,579 disease concepts (2021 version of the DO); (2) including more exquisite relationship with weights between entities for knowledge graph construction. This significant expansion of the number of diseases represents diseases across all disease types; (3) all systems [12, 15, 17, 30, 31, 32] described above are designed for human consumption only, whereas the diseasomics presented here is both human understandable and machine interpretable; (4) because the diseasomics knowledge is machine interpretable, other ontology based knowledge sources or knowledge extracted using AI techniques can easily be integrated semantically or thematically. Supplementary Information SI3 shows how diseasomics is integrated with spatial comorbidity, resistomics, patholomics, oncolomics, EHR, and a secured telemedicine system. (5) diseasomics is stored in a graph database and can be accessed through API (Application Programming Interface) for the use of a health workers at the point-of-care.

**Reviewer #2:**
- Although I'm not an expert in the technical side of NLP methods, I think any results should be presented with the current benchmarks. The paper does not seem to provide these. Related to the evaluation, the paper states that "We randomly generated 100 two co-occurring symptoms combination and 100 three co-occurring symptoms combination." what's the reasoning behind these numbers?

**>>>> Authors' Response**

We validated the diseasomics knowledge graph using multiple approaches as following:

Approach#1) Under the supervision of experienced clinician and author Prof Dr Rakesh Biswas (RB), MD, 200+ common infectious and non-communicable diseases were identified. The symptoms for these common diseases were extracted from the diseasomics KG and validated by 5 junior clinicians under RB's supervision. These clinicians were allowed to refer to textbooks and Google search.

Approach#2) In this approach, we conceptually used the Monte Carlo (MC) methods for 50 random draws of two symptoms and 50 random draws of three symptoms. MC methods are a class of computational algorithms that rely on repeated random sampling to compute quantities of interest. These 100 combinations of symptoms (50 two symptoms + 50 three symptoms) were given to three certified clinicians for blind validation in a city near National Institute of Technology Karnataka, Surathkal, India without sharing the associated diseases in the diseasomics KG. Same list of 100 symptoms combinations were also given to three health workers (two certified dentists and one certified nurse) who are pursuing MBA in Hospital and Healthcare Management at Pune, India for blind validation. These six volunteers were allowed to refer to Google search engine and literature. The variability between the volunteers were very high. This was mainly due to the subjectivity associated with the interpretation of symptoms. Due to high variability we failed to statistically quantify the results with respect to the diseasomics KG. This type of wide variability is not new at primary care – a study found that second opinion discovered 88% misdiagnosis at the primary care [50].

Approach#3) Because of the failure of blind validation in Approach#2, we used MC technique differently. In this approach, we doubled the number of Monte Carlo trials from 50 to 100 and extracted the associated diseases from the KG. MC methods are suitable when it is infeasible to compute an exact result with a deterministic algorithm [51]. The use of MC techniques in the field of medical physics and

particle therapy have been increasing where high accuracy are crucial elements for a successful planning of patient treatment and its verification [52]. We randomly generated 100 two co-occurring symptoms combinations and 100 three co-occurring symptoms combinations. We used these 200 co-occurring symptoms combinations to fetch the associated 890 diseases from our diseasomics KG as described in the paper. This 890 disease-list was used by an experienced clinician and authors Prof Arnab Ghosh (AB), MD, DNB, PhD scholar for manual validation. The result of this approach is reported in the main text and provided in SI2. AB's results are based on experience and validated through text books of medicine like Harrison's Principles of Internal Medicine, Bailey and Love's Test book of surgery etc. The results have been reviewed by experienced clinician and author Prof Prantar Charkrabarty (PC), MD, DNB, DM. PC has contributed substantially towards medical research, medical education and challenges in Primary, Rural, Remote care, and hematology at the national level.

Approach#4) In addition to approaches #1, #2, and #3 as described above, the diseasomics Knowledge Graph is available for open access at the URL "https://triage.cyberneticcare.com/diseasePrediction" for general use. This Web application accesses the KG through API. The diseasomics knowledge graph (KG) is currently being tried by experienced clinicians in India and Germany.

**Reviewer #2:**
- Mobile app part of the paper is very underdeveloped and I think there is no need to include it without any evaluation.

>>>> **Authors' Response**
We agree and have removed the whole section on mobile phone

---------------
## Reviewer#3
---------------
**Reviewer #3:**
The introduction was too broad and nonspecific, particularly for this targeted journal and audience. Much more time should have been spent discussing previous work around differential diagnosis support systems (e.g. DXplain).

>>>> **Authors' Response**
Thank you for this constructive review. The introduction has been revised and augmented with a more detailed discussion of previous differential diagnosis systems.

We replaced the paragraph in Page 2
[Original Text, old reference numbering]
Medical decision making is a complex process and can be viewed along a spectrum, with categorical (or deterministic) reasoning at one extreme and probabilistic (or evidential) reasoning at the other extreme [10]. Categorical medical decision-making processes follow single flowchart, whereas, probabilistic decision making involves multiple overlapping flowcharts with added uncertainties. There were various attempts to build accurate clinical decision support systems (CDSS) helping in diagnoses [11]. Many AI systems have been built with a focus on machine interpretable medical knowledge and medical decision making [7, 12, 13, 14] such as diagnostic decision support systems (DDSS) [11, 15, 16]. From a knowledge management perspective Barabasi constructed an early version of a bipartite diseasome network [14]. Diseasomics presented here is based on a multipartite graph that combines biomedical ontologies, knowledge from published data, literature, and EHR to provide a digital twin of the disease knowledge. Diseasomics offers a comprehensive machine interpretable disease knowledge

that combines symptoms ontology (SO), disease ontology (DO) [17], SNOMED CT (Systematized Nomenclature of Medicine -- Clinical Terms) [18, 19], UMLS CUI (Unified Medical Language System, Concept Unique Identifier) [20], ICD10 CM (International Classification of Diseases 10th Revision) [21], HGNC (Human Genome Organization Gene Nomenclature Committee) [22], genes, pharmacogenomics (PharmGKB) [23], and knowledge from patient data from emergency room, spatial disease comorbidity, and temporal disease comorbidities that can be used to allow a machine to make medical decisions (Figure 1). These rich knowledge bases of biomedical data use their own coding systems that are not interoperable, thus making it difficult for non-specialists to use them at the point-of-care. Diseasomics has removed this constraint and has made the knowledge graph coding system agnostic.

With the following new text:
[New Text, new reference numbering]
Medical decision making is a complex process and can be viewed along a spectrum, with categorical (or deterministic) reasoning at one extreme and probabilistic (or evidential) reasoning at the other extreme [8]. Categorical medical decision-making processes follow single flowchart, whereas, probabilistic decision making involves multiple overlapping flowcharts with added uncertainties. Numerous diagnostic decision, clinical decision and differential diagnosis support systems address this complexity, aiming to provide effective tools to guide disease characterization, enhance patient safety through a reduction medication errors, improve clinical guidelines adherence, and identify relevant imaging analysis based on the patient's symptoms matched to diagnoses [9, 10, 11]. Tools are developed both commercially (e.g.visualDx [10, 12, 13, 14], AMBOSS [15], Ada Health [16]) and in clinical laboratories (e.g. Dxplain [17, 18]) to integrate medical images, explore potential etiologies in literature or characterize electronic medical textbooks for sets of human diseases. Artificial Intelligence (AI) information systems provide novel tools in the diagnostician's toolkit, towards more informed clinical decisions and broadening the availability of diagnosis support in rural communities [19, 20].

To address the challenges of offering an interoperable, openly available differential diagnosis tool across the breadth of human diseases, we have developed the 'Diseasomics' tool that combines biomedical ontologies, knowledge from published data and EHR records to provide a disease knowledge, pharmacogenomic knowledge bases regularized and connected via biomedical ontologies and controlled vocabularies. The Symptom Ontology (SYMP), Human Disease Ontology (DO) [21], SNOMED CT (Systematized Nomenclature of Medicine – Clinical Terms) [22, 23], UMLS CUI (Unified Medical Language System, Concept Unique Identifier) [24], ICD10 CM (International Classification of Diseases 10$^{th}$ Revision) [25], HGNC (Human Genome Organization Gene Nomenclature Committee) [26], genes, and pharmacogenomics (PharmGKB) [27] enable interoperability and foster reuse across diagnostic systems.

**Reviewer #3:**
The validation of the tool was particularly suspect. It would be much more useful to be validated against clinical opinion and practice rather than itself. As a specific tool for untrained providers it might be useful, but certainly not for trained clinicians.

**>>>> Authors' Response**

We validated the diseasomics knowledge graph (KG) using multiple approaches as following:

Approach#1) Under the supervision of experienced clinician and author Prof Dr Rakesh Biswas (RB), MD, 200+ common infectious and non-communicable diseases were identified. The symptoms for these

common diseases were extracted from the diseasomics KG and validated by 5 junior clinicians under RB's supervision. These clinicians were allowed to refer to textbooks and Google search.

Approach#2) In this approach, we conceptually used the Monte Carlo (MC) methods for 50 random draws of two symptoms and 50 random draws of three symptoms. MC methods are a class of computational algorithms that rely on repeated random sampling to compute quantities of interest. These 100 combinations of symptoms (50 two symptoms + 50 three symptoms) were given to three certified clinicians for blind validation in a city near National Institute of Technology Karnataka, Surathkal, India without sharing the associated diseases in the diseasomics KG. Same list of 100 symptoms combinations were also given to three health workers (two certified dentists and one certified nurse) who are pursuing MBA in Hospital and Healthcare Management at Pune, India for blind validation. These six volunteers were allowed to refer to Google search engine and literature. The variability between the volunteers were very high. This was mainly due to the subjectivity associated with the interpretation of symptoms. Due to high variability we failed to statistically quantify the results with respect to the diseasomics KG. This type of wide variability is not new at primary care – a study found that second opinion discovered 88% misdiagnosis at the primary care [50].

Approach#3) Because of the failure of blind validation in Approach#2, we used MC technique differently. In this approach, we doubled the number of Monte Carlo trials from 50 to 100 and extracted the associated diseases from the KG. MC methods are suitable when it is infeasible to compute an exact result with a deterministic algorithm [51]. The use of MC techniques in the field of medical physics and particle therapy have been increasing where high accuracy are crucial elements for a successful planning of patient treatment and its verification [52]. We randomly generated 100 two co-occurring symptoms combinations and 100 three co-occurring symptoms combinations. We used these 200 co-occurring symptoms combinations to fetch the associated 890 diseases from our diseasomics KG as described in the paper. This 890 disease-list was used by an experienced clinician and authors Prof Arnab Ghosh (AB), MD, DNB, PhD scholar for manual validation. The result of this approach is reported in the main text and provided in SI2. AB's results are based on experience and validated through text books of medicine like Harrison's Principles of Internal Medicine, Bailey and Love's Test book of surgery etc. For the purpose of provenance of the 90.45% accuracy, we have added three additional columns in the SI2 table. These fields are 'Reference', 'Remarks', and 'Description'.The results have been reviewed by experienced clinician and author Prof Prantar Charkrabarty (PC), MD, DNB, DM. PC has contributed substantially towards medical research, medical education and challenges in Primary, Rural, Remote care, and hematology at the national level.

Approach#4) In addition to approaches #1, #2, and #3 as described above, the diseasomics Knowledge Graph is available for open access at the URL "https://triage.cyberneticcare.com/diseasePrediction" for general use. This Web application accesses the KG through API. The diseasomics knowledge graph is currently being tried by experienced clinicians in India and Germany.
