# Supplementary material for: Diseasomics: Actionable machine interpretable disease knowledge at the point-of-care
Source: PLOS Digit Health. 2022 Oct 20;1(10):e0000128. doi: 10.1371/journal.pdig.0000128 (PMC9931276; doi:10.1371/journal.pdig.0000128)
Supplement: Attachment — Submitted filename: Response to Reviewers.pdf [file pdig.0000128.s007.pdf]

Response to Reviewer
Apr 5, 2022

Reviewer:
- The knowledge graph and associated software do not appear to be available in an open source repository. The Data Availability statement says that "The NeoImportDO.zip self-loading knowledge graph can be downloaded from “https://cyberneticcare.com", but it is unclear where on this website the data can be downloaded from and I would not consider this a suitable long term repository. Instead, I suggest the data and software should be deposited in a repository such as Zenodo or Figshare with a formal citation and persistent link (DOI).

Authors' Response >>> We have created a repository on Zenodo.org. The NeoImportDO.zip file can be downloaded from there. A DOI [58] was created that is formally cited in the reference list.

Reviewer:
- There is a demo of the prediction tool at: https://triage.cyberneticcare.com/diseasePrediction but there appears to be no documentation and I was unable to successfully generate a prediction using the tool.

Authors' Response >>> We have revised the https://triage.cyberneticcare.com/diseasePrediction Web page. We have added examples to avoid any confusion. A documentation page has also been included and can be accessed by clicking the [Read more ...] link. The "Description" column in the "Predicted Disease" contains Web links. These links can be used for validation, evidence, and further details.

Reviewer:
- Given that model validation is perhaps the most important aspect of the paper, I think significantly more effort is needed in this area. Currently the validation is described at a very high level, with limited details of the approach and results.
- In addition, the language in the validation section often lacks clarity (e.g. it is unclear to me exactly what is meant by "Monte Carlo methods are a class of computational algorithms that rely on repeated random sampling to compute quantities of interest without bias"...presumably "compute quantities" means estimating parameters? An unbiased estimate of a parameter requires an unbiased method to estimate the parameter -- using MC is not sufficient).

Authors' Response >>>
We are now describing the process of validation in greater detail as requested and replaced the entire section with the following text:

We performed the validation of the diseasomics knowledge graph in different ways. For validation of SNOMED CT, Trajectory, and DisGeNET we had selected about 50 nodes randomly and tested them manually one by one.

For differential diagnosis validation, we used symptoms as listed in the symptom ontology file symp.obo. We used MetaMap [41] to get the UMLS CUI codes for all symptoms in symp.obo. This gave us 65 different types of UMLS codes like [Amino Acid, Peptide, or Protein, Enzyme], [Biomedical or Dental Material], [Body Location or Region], [Body System], [Cell], [Pathologic Function], [Sign or Symptom], [Tissue] etc. From these UMLS codes we selected only those UMLS CUIs codes that map to 'Sign or Symptom', which gave us 240 ‘Sign or Symptom’ that were used for validation.

In our first approach, we performed 50 random draws of two symptoms and 50 random draws of three symptoms. These 100 combinations of symptoms (50 two symptoms + 50 three symptoms) were given to three certified clinicians for blind validation in a city near National Institute of Technology Karnataka, Surathkal, India without sharing the associated diseases in the diseasomics knowledge graph. All these three clinicians had 2 years of experience following their MBBS medical training and compulsory rotating internship in the hospital. The same list of 100 symptoms combinations were also given to three health workers (two certified dentists and one certified nurse) who are pursuing MBA in Hospital and Healthcare Management at Pune, India for validation in a blinded manner. In the process of blinded validation, all the six healthcare professionals were requested to “make a list of the possible differential diagnoses for 100 virtual patients who presented with these symptom combinations". These six volunteers were allowed to refer to Google search engine and published literature. The variability between the results generated by the volunteers was very high. This was mainly due to the subjectivity associated with the interpretation of symptoms. Due to a very high variability, we failed to statistically quantify the results with respect to the diseasomics knowledge graph. This type of wide variability has been well documented at the primary care level – a study at Mayo Clinic found that second opinion discovered 88% misdiagnoses at the primary care [50].

Since we were not successful in quantifying the results with young healthcare providers in our first approach, we then attempted a validation with experienced tertiary care doctors and authors Dr Arnab Ghosh, MD, DNB, MNAMS, PhD scholar, and Dr Prantar Chakrabarti, MD, DM, DNB in the second approach. We doubled the number of random draws from 50 to 100 symptom combinations and extracted the associated diseases from the knowledge graph.

We randomly generated 100 two co-occurring symptoms combinations and 100 three co-occurring symptoms combination. We used these 200 co-occurring symptoms combinations to fetch the associated diseases from our knowledge graph. For two symptoms, random 30 combinations (out of 100) did not produce any result. Similarly, for three symptoms 45 combinations (out of 100) did not produce any result. The 70 double-symptom combination fetched 751 diseases and 55 triple-symptom combinations fetched 139 diseases. This 890 disease-list was used for manual validation. We considered these 890 instances that fetched disease from knowledge graph as positive cases and 75 instances that did not fetch any associated disease from the knowledge graph as negative cases.

Out of 751 positive double-symptom diseases, the machine could correctly make differential diagnoses in 674 cases; and, 77 incorrect interpretations were observed. Out of 139 positive cases of three symptoms, 131 cases were correctly diagnosed, and 8 cases were incorrect. This gave us 805 True-positive (TP) and 85 False-positive (FP) cases. The machine’s diagnoses were marked wrong considering some conditions to be having random associations for example muscle pain with fever. Therefore, though the machine identified the association correctly, we labeled it “incorrect” considering a casual co-existence of symptoms. We labeled 9 out of 85 such observations “incorrect” giving benefit of doubt to the manual system of evaluation. Out of the 75 negative cases 64 combinations should produce some diagnosis which are missing in the knowledge graph. 11 combinations of negative cases do not have any known disease association in literature. This gave us 64 False-negative (FN) and 11 (TN) True-negative. Table 4 summarizes the differential diagnosis results. The results are available in SI2.xls.

Table 4: Results of the differential diagnosis 2- and 3-symptom predictions

**Symptom combination is associated with a disease**	**DDx Correct**	**DDx Incorrect**

Positive	805 (TP)	85 (FP)
Negative	11 (TN)	64 (FN)

Based on the table (4) above we computed the overall accuracy and the F1 score:

The overall accuracy is: [(TP+TN)/(TP+TN+FP+FN)] = 0.84559
The F1 Score computes to: [2*TP /(2*TP + FP + FN)] = 0.91529

Symptom checkers and differential diagnostic tools are typically evaluated on the basis of clinical vignettes or patient cases with confirmed and known diagnoses [51, 52]. Therefore, in addition to the random combination of symptoms we performed a third validation cycle based on given diseases and their symptoms. We randomly selected 25 diseases including COVID19 from a set of typical diseases presented to general practitioners across different geographies and listed all known associated symptoms as UMLS codes (see supplementary file SI3.xlsx). These symptoms were verified by Dr. Puja Chowdhury. One by one, the symptom sets were fed into the knowledge graph-based diagnostic tool for validation. When a disease was associated with a maximum of 3 symptoms we used all symptoms for differential diagnosis. In case of more than 10 symptoms, we used a random selection of 10 symptoms to construct a symptom set that could be used for validation. The details are given in supplementary file SI3.xlsx.

Reviewer:
- Making the code and data used in the model analysis/validation available in a public repository would be very helpful. As far as possible, these resources should be organised in a way that demonstrates the approaches and allows them to be reproduced.

Authors' Response >>> The pipeline comprises of semantic integration of ontologies, and manually curated data by subject matter experts. Ontologies are thematically integrated with cross sectional, and longitudinal statistically significant data from EHR. The pipeline used many open domain and proprietary licensed tools and manual techniques that is described in the Methods section. All the open data and curated open data are embedded in the zip file NeoImportDO.zip. The semantic integration of ontologies is automated and reproducible through the source code embedded within the zip file NeoImportDO.zip. The thematic integration of statistically significant EHR data is also automated and source code for reproducibility is embedded in the zip file. The source code to load and create the knowledge graph from scratch is embedded within the zip file as well. The code within the zip file is to best of our understanding is reproducible and can be validated. The results in supporting information "SI2.xlsx" now includes a column "Reference" (Col#H), which is in fact the provenance for validation. This is, to best of our understanding is also reproducible and can be validated.
