# Supplementary material for: Diseasomics: Actionable machine interpretable disease knowledge at the point-of-care
Source: PLOS Digit Health. 2022 Oct 20;1(10):e0000128. doi: 10.1371/journal.pdig.0000128 (PMC9931276; doi:10.1371/journal.pdig.0000128)
Supplement: Attachment — Submitted filename: Response to Reviewers.pdf [file pdig.0000128.s008.pdf]

Response to Reviewers and Editor

August 8, 2022

Journal Requirements:

1. Please review your reference list to ensure that it is complete and correct. If you have cited papers that have been retracted, please include the rationale for doing so in the manuscript text, or remove these references and replace them with relevant current references. Any changes to the reference list should be mentioned in the rebuttal letter that accompanies your revised manuscript. If you need to cite a retracted article, indicate the article's retracted status in the References list and also include a citation and full reference for the retraction notice.
>>>> Authors' Response
We have checked the reference to ensure that none of the reference entries are retracted.

Additional Editor Comments (if provided):

Thanks for responding to the points raised by reviewers. On reading the updated version, I think it is important that the limitations and potential risks of the tool should be considered.
>>>> Author Response
We have updated the Abstract and Conclusion as suggested. We have also updated the presentation tool to overcome ordering of the diseases.

(1) clarifying in the abstract that there are important limitations of the knowledge network
>>>> Authors' Response
We added the following text:

The machine interpretable knowledge graphs presented in this paper are associations between various entities and do not imply causation. Our differential diagnostic tool focusses on signs and symptoms and does not include a complete assessment of patient's lifestyle and health history which would typically be necessary to rule out conditions and to arrive at a final diagnosis. The predicted diseases are ordered according to the specific disease burden in South Asia. The knowledge graphs and the tools presented here can be used as a guide.

(2) explicitly discuss limitations and risks in the discussion section, and how these might be mitigated.
>>>> Authors' Response
We have added the following text:

Clinical diagnosis involves the discovery of the causation of the illness. The machine interpretable knowledge graphs presented in this paper are associations between various conditions and do not imply causation. The knowledge graphs and the associated tools presented here can be used as a guide. This will be suitable for triaging and decision support about when it is a good time to seek medical help or a referral.

These tools are also effective as a search engine for disorders that share similar symptoms, e.g., it is very easy to produce a list of all diseases that share fever as a common symptom. The knowledge graph can also

be used as a tool in medical studies and nursing training to compile rare diseases associated with a set of symptoms that could potentially be overlooked if the healthcare provider does not have the special experience. The knowledge graph produces a list of diseases that is ordered by prevalence/disease burden from South Asia. This can lead to confusions if life threatening diseases like rabies appear on the list that are very rare in Central Europe but unfortunately quite common in India. Future versions of our knowledge graph will be customized to produce a list of potential diseases ordered by their prevalence in other regions including Europe and the US.

Clinical diagnosis is a very complex process. Tools like the knowledge graphs presented here can assist and support the differential diagnostic process but do not replace the careful root cause analysis, elimination process, and gathering of further information about lifestyle and health history.

For example, if I search for "fever" at https://triage.cyberneticcare.com/diseasePrediction, the first result is "Rabies". What are the negative implications of offering (most likely) incorrect predictions?
>>>> Authors' Response
For the differential diagnosis tool, we fetch the knowledge from the knowledge graph and present it as it arranged in the ontologies. This presentation is arbitrary and does not have any logical order. To overcome this limitation, we have enhanced the tool to order the output from the knowledge graph such that the most likely diseases are presented at the top of the list.

[Note: HTML markup is below. Please do not edit.]

Reviewers' comments:

Reviewer's Responses to Questions

Comments to the Author
1. If the authors have adequately addressed your comments raised in a previous round of review and you feel that this manuscript is now acceptable for publication, you may indicate that here to bypass the "Comments to the Author" section, enter your conflict of interest statement in the "Confidential to Editor" section, and submit your "Accept" recommendation.

Reviewer #1: All comments have been addressed
>>>> Authors' Response: Thank you

----

2. Does this manuscript meet PLOS Digital Health's publication criteria? Is the manuscript technically sound, and do the data support the conclusions? The manuscript must describe methodologically and ethically rigorous research with conclusions that are appropriately drawn based on the data presented.

Reviewer #1: Yes
>>>> Authors' Response: Thank you
----

3. Has the statistical analysis been performed appropriately and rigorously?

Reviewer #1: Yes
>>>> Authors' Response: Thank you
----

4. Have the authors made all data underlying the findings in their manuscript fully available (please refer to the Data Availability Statement at the start of the manuscript PDF file)?

 The PLOS Data policy requires authors to make all data underlying the findings described in their manuscript fully available without restriction, with rare exception. The data should be provided as part of the manuscript or its supporting information, or deposited to a public repository. For example, in addition to summary statistics, the data points behind means, medians and variance measures should be available. If there are restrictions on publicly sharing data—e.g. participant privacy or use of data from a third party—those must be specified.

Reviewer #1: Yes
>>>> Authors' Response: Thank you

----

5. Is the manuscript presented in an intelligible fashion and written in standard English?PLOS Digital Health does not copyedit accepted manuscripts, so the language in submitted articles must be clear, correct, and unambiguous. Any typographical or grammatical errors should be corrected at revision, so please note any specific errors here.

Reviewer #1: Yes
>>>> Authors' Response: Thank you
----

6. Review Comments to the Author
Please use the space provided to explain your answers to the questions above. You may also include additional comments for the author, including concerns about dual publication, research ethics, or publication ethics. (Please upload your review as an attachment if it exceeds 20,000 characters)

Reviewer #1: Clinical diagnosis is a complex process based not solely on symptoms but on signs and symptoms, paraclinical studies and physical exam. However the combination of resources and the model presented here is impressive. There have been earlier attempts based also on the UMLS and Bayes (Homero, and a couple other systems), abandoned from clinical use because of it was not specific enough and clinicians do not really need general diagnosis tools like this. It would be best to promote it as a layman's tool to decide when is a good time to seek medical help.
All comments were addressed properly.
>>>> Authors' Response: Thank you
----

7. PLOS authors have the option to publish the peer review history of their article (what does this mean?). If published, this will include your full peer review and any attached files.

Do you want your identity to be public for this peer review?
If you choose “no”, your identity will remain anonymous but your review may still be made public.

For information about this choice, including consent withdrawal, please see our Privacy Policy.

Reviewer #1: Yes: Laritza M Rodriguez MD, PhD
>>>> Authors’ Response: Thank you
